# Which horticultural activities are more effective for children’s recovery from stress and mental fatigue? A quasi-experimental study

**DOI:** 10.3389/fpsyg.2024.1352186

**Published:** 2024-04-12

**Authors:** Le Guo, Wei Xu, Yuyi Shi, Shuguang Gao, Chengxiang Xiao, Xiaoxiao Zhang, Xifan Liu, Qingyu Zhang, Yanlong Zhang

**Affiliations:** ^1^College of Landscape Architecture and Art, Northwest A&F University, Xianyang, China; ^2^School of Biological and Environmental Engineering, Xi’an University, Xi’an, Shaanxi, China

**Keywords:** horticultural therapy, electrocardiogram, electroencephalogram, psychological questionnaire, health status

## Abstract

**Introduction:**

Studies have established the benefits of horticultural therapy and activities for human health and well-being. Nonetheless, limited research has been conducted on the potential restorative advantages and distinctions between different types of horticultural activities in terms of stress reduction.

**Methods:**

This study employed a quantitative research method to investigate the stress recovery benefits of five horticultural activities (flower arrangement, sowing and transplanting seeds, *kokedama* crafting, pressed flower card making, and decorative bottle painting with dried flowers) and one reference activity (short composition writing) for children. The experiment was conducted in a children’s activity center’s multi-purpose classroom with 48 elementary students aged 9–12 years. The subjects first took a stress test to induce stress and then engaged in horticultural activities for 20 min. Physiological stress was assessed using electrocardiograms and electroencephalograms as feedback indicators. Psychological and emotional changes were determined using the Positive and Negative Affect Schedule for Children and Self-Assessment Manikin scales.

**Results:**

The results demonstrated that horticultural activities greatly reduced physiological fatigue, and their recovery benefits were significantly greater than those of the reference activity. The recovery effects from different horticultural activities were similar across physiological indicators, although flower arrangement and sowing and transplanting seeds exhibited relatively robust recovery benefits. The heart rate and α-EEG-based generalized estimating equation revealed that horticultural activities offered significantly better relative recovery at each time phase of operation than the reference activity, with girls showing a 3.68% higher relative recovery value than boys. Flower arrangement and *kokedama* crafting offered better physiological recovery for students with prior horticultural experience, and these two activities received the highest scores in terms of positive effects and the “pleasure” dimension. Students believed that participating in horticultural activities resulted in a noteworthy increase in personal confidence and a greater sense of achievement.

**Conclusion:**

The study suggests that horticultural activities that involve real and vibrant plants or natural materials and are more attractive have more stress-relieving benefits. We conclude that horticultural activities are beneficial leisure activities that aid in stress relief for children and that it is important to consider the attributes of activities when developing horticultural programs for elementary students.

## 1 Introduction

Children are a vulnerable social group, and their lives, leisure, proactive perceptual abilities, and behavior have been adversely impacted by rapid urbanization and industrialization processes (Weber and Stickl, 1982). Children are now spending less time outdoors and in nature (Soga et al., 2018). Consequently, Richard (2008) argued that they are prevented from freely exploring the “wild” and proposed the notion of “nature deficit disorder.” The limitation of children’s engagement in outdoor and natural activities has deepened their disconnect from nature and reduced their attention to it (Chawla, 2020; Xu and Jiang, 2022). Children who are disconnected from nature and spend more time indoors tend to have poorer health and a lower quality of life and well-being (Roberts et al., 2020). The presence of green spaces and the level of physical activity and time spent outdoors are positively related (Zare Sakhvidi et al., 2023). A longitudinal study found that boys living in areas with 20% green space get 55 min less physical activity per week than those living in areas with 50% green space (Sanders et al., 2015). The prevalence of sedentary lifestyles among children is steadily increasing, and insufficient physical activity increases health risks (Boreham and Riddoch, 2001; Garwood and Lindmeier, 2019). Another study reported that children spend nearly 76% of their leisure time watching TV, using the computer, or doing homework (Strauss et al., 2001). Studies have shown a positive correlation between depressive symptoms and screen time among adolescents (Hoare et al., 2016). Furthermore, children’s experiences of physical activity and nature can influence their motivation to participate in activities as adults (Hovell et al., 1991), which is becoming increasingly evident in terms of both direct and long-term effects on children (Strong et al., 2005). Early-formed behavior patterns can carry into adolescence and adulthood (Biddle et al., 2010). A retrospective study conducted by Nancy and Kristi (2006) found a positive correlation between childhood participation in “wild nature” nature and environmental attitudes in adulthood, making adults more likely to embrace environmentalism.

The positive effects of nature on physical and mental health have been widely confirmed (Ulrich et al., 1991; Kaplan, 1995; Van den Bogerd et al., 2020). Horticultural therapy has emerged as a form of nature-assisted therapy (Berger and McLeod, 2006), becoming a complementary treatment method to traditional medicine. In recent years, horticultural activities have emerged as a low-cost leisure activity (Ainamani et al., 2022). Daily horticultural activities, which are fun, spontaneous, and healthy, can have effects similar to horticultural therapy (Li, 2011): Participating in horticultural-related activities can improve physical functions (increase physical activity, regulate limb coordination) and provide individuals with emotion regulation, improvements in cognition, increased social interactions, and enhanced well-being (Chalmin-Pui et al., 2021; Watanabe et al., 2021; Kim et al., 2022). Hawkins et al. (2011) suggest that horticultural activities possess stress-buffering properties as they offer opportunities for contact with nature. Generally, people are consistently exposed to stress, and prolonged or intermittent stress can have lasting detrimental effects on physiological functions (Anisman and Merali, 1999). The most common type of intervention to study the effects of horticultural activities on stress and psychological states is programs—usually of short (less than 10 sessions) or medium (11 to 20 sessions) duration (Park et al., 2016). The study subjects generally fall into one of the three categories as follows. (1) Older adults: These individuals are often susceptible to diseases, psychological issues, and social circumstances and frequently experience worry, depression, and loneliness (Nicholas et al., 2019). Several randomized controlled trials have demonstrated that compared to the control group, the old adults in the horticultural intervention group showed a significant decrease in perceived stress and significantly lower levels of salivary cortisol than at baseline (Han et al., 2018; Chan et al., 2022; Lee et al., 2022). (2) Students and working individuals: They may experience stress from personal concerns, academics, work, and neighborhood risks like violence and disease (WHO, 2020). According to Chen et al. (2015), Odeh et al. (2022), and Han (2017), horticultural programs improved work-related stress responses and overall health in working staff, reduced trait anxiety levels in healthy women in the horticultural activity group more effectively than in those in the art creation group, and resulted in greater self-reported stress alleviation in high school students actively caring for plants compared to passively exposed students. Domestic gardening even enhanced stress resilience during the COVID-19 pandemic (Sia et al., 2022). (3) People with mental illnesses or psychological trauma: Among prisoners, post-war veterans, people who experience postpartum depression, and disaster survivors, horticultural therapy interventions improved factors related to suicide ideation and behavior hazards like negative emotions, stress, despair, and loneliness (Kotozaki, 2014, 2020; Lee et al., 2021; Meore et al., 2021). Aside from longitudinal studies, research has also explored the immediate health effects of engaging in a single horticultural activity, typically lasting for 3–20 min (Hassan et al., 2018, 2019b). Although horticultural activity has been shown to alleviate stress, sadness, anxiety, and other negative emotions in different groups, studies have also reported some inconsistencies (Du et al., 2022). Furthermore, there is a clear gap in research regarding the effects of these interventions on children, indicating the need for empirical studies determining the efficacy of horticultural interventions in aiding children’s recovery from psychological and physiological stress. Lu et al. (2023) also noted that the stress-relieving effects of horticultural activities are more pronounced in people aged over 60 years.

Contemporary youngsters also encounter a multitude of pressures. Their primary challenges are academic pressure and performance, especially for Chinese children (Liang, 2018). Beyond competition for academic success, other implicit stressors (e.g., boring curricula, stage fright, disruptive students) that affect children may be ignored (Matheny et al., 1993). Therefore, whether horticultural activity can alleviate elementary students’ stress and mental fatigue in response to antecedent stressors (e.g., classroom tests, teacher criticism, conflict with peers) remains uncertain. Furthermore, the use of different horticultural activities as interventions for specific social groups has yielded inconsistent results for stress, psychophysical states, and engagement (Gigliotti et al., 2004; Lee et al., 2018; Tu et al., 2020). As no related research has yet been conducted with the general child population, the following points have not been addressed: whether different types of horticultural activities have diverse stress recovery effects on this population, which activity is more effective for students’ recovery, and what the relation between activity preferences and improvements in physiological stress is.

We prioritized indoor horticultural activities, considering the ease of implementing them during daily classes and controlling for confounding factors. Moreover, evidence shows that actively gardening is more emotionally rewarding than passive scenic-viewing activities (Ambrose et al., 2020). Thus, we targeted five indoor, hands-on horticultural activities: flower arrangement (FA), *kokedama* crafting (which translates literally “moss ball” crafting; KC), sowing and transplanting seedlings (SATS), pressed flower card-making (PFCM), and decorative bottle painting with dried flowers (DBP). We used psycho-physiological responses to explore the impact of different horticultural activity types on elementary students’ physiological arousal and emotions. Our study aimed to ascertain the potential of horticultural activities for promoting stress recovery and well-being among this group, while also providing a scientific basis and theoretical foundation for implementing and promoting horticultural curriculum in elementary schools. Specifically, we aimed to address the following research questions:

1. What physiological impact do different types of horticultural activities have on pre-stressors in elementary students?
2. What emotional and affective impacts do different types of horticultural activities have on pre-stressors in elementary students?
3. Do elementary students’ stress recovery levels show physiological differences when engaging in different types of horticultural activities, and do they have different subjective experiences toward different activities in terms of psychology and emotions?
4. How do interactions between physiological feedback and time change during the activity while controlling for sociodemographic factors?

## 2 Materials and methods

### 2.1 Participant enrollment

The research team visited 15 elementary schools in the Yangling Agricultural Hi-Tech Industries Demonstration Zone (Shaanxi, Xianyang, China) in mid-March 2022 to identify study partners. Six principals agreed to distribute recruitment messages within their schools. Class teachers then posted electronic recruitment posters in the student guardians’ information exchange group on the WeChat mobile application to call for participants. Guardians could also share these posters with acquaintances. Students interested in participating could have their parents contact the research assistant using the contact information provided in the poster to acquire specific details about the activities. Considering the practical abilities and comprehension of elementary students, we set the following inclusion criteria: third- to sixth-graders with normal vision and hearing; without allergies to fresh-cut flowers; and without physiological, emotional, or latent heart diseases.

We used G*Power (3.1.9.4) to calculate the sample sizes for paired *t*-tests, one-way analysis of variance (ANOVA), and repeated-measures analysis with *p*-values and power values of 0.05 and 0.80, respectively (Buchner et al., 2023). Paired sample *t*-tests and one-way ANOVA with effect sizes of 0.50 and 0.25 required 34 and 36 participants, respectively. With a partial η ^2^ of 0.06, six groups, and four measures, we determined that 42 participants were required for repeated measures analysis. All analyses used a medium effect size.

The participant recruitment process lasted about 3 weeks, and a total of 55 primary students signed up by the recruitment deadline. Some students withdrew for personal reasons during the experimental phase; ultimately, 48 completed the experiment (27 girls, mean age = 10.42 years, SD = 1.05). The study was approved by the Ethics Committee of Northwest A&F University.

### 2.2 Place and time

The experiment was conducted in the summer mid-semester (April–June 2022) when elementary students were in an ordinary academic period and their mental state was not negatively affected by pressures from approaching final exams. To faithfully replicate the classroom scenes, we employed a quasi-experimental mode. The experiment site was selected as a multi-purpose classroom at the Children’s Cultural Activity Center in Shaanxi. We controlled for the trial site’s environmental factors considering that views, lighting, and thermal conditions can affect indoor occupants’ cognitive performance (Ko et al., 2020). The classroom doors and windows were closed, curtains were drawn, and the central air conditioning’s dehumidification mode and LED educational lighting were turned on 30 min before the daily activity to control for confounding factors in the environment. The indoor temperature, relative humidity, and illumination were maintained at approximately 25°C, 55%, and 640 lux, respectively.

### 2.3 Course contents and arrangement

The activities were conducted as horticultural-themed courses from Monday to Saturday in the afternoons after school, with each day featuring a themed activity. Our study employed a within-subject design, and six elementary students took all courses in each session. Students participated in the daily theme activities in two-person groups. The order of group activities each day followed a rotation. After three courses, the members of each group were reorganized to ensure that each participant in the group was unfamiliar with the other. Horticultural classes were randomized before each session, and the order was not disclosed to participants beforehand. The participants were also advised to refrain from engaging in activities similar to those in this study.

We referred to the horticultural activities classification method proposed by Park et al. (2016), excluding “outdoor gardening,” and selected FA, KC, SATS, PFCM, and DBP as the representative activities for the “flower arranging art,” “live plant crafts,” “indoor planting,” “art or pressed flower crafts,” and “other activities (including food making, art, exhibitions, etc.)” categories. Each activity had unique characteristics and objectives (Table 1): The SATS and KC activities were focused on horticultural techniques, whereas the other activities were more inclined toward artistic creation. Further, the textures and color tones of the plant materials were distinctive, providing unique sensory experiences for every activity. We used free composition writing (CW), a typical classroom activity, as a reference activity. The content of the compositions was based on the knowledge and skills the participants had acquired from the course and their varied preferences for activities.

**Table 1:** Horticultural activities theme, contents, materials, and objectives.

Theme	Contents	Materials and tools	Objectives
Sowing and transplanting seedings (SATS)	① PPT presentation on seed morphology; Key points for seedling care and management; ② Mixing soil, watering, planting seedlings, etc.	Mini sunflower seeds, edible lemon mint seedlings, cultivation soil, Nutrient-rich substrate, PE cultivation pot, watering can, plant labels	① Learn plant reproduction methods, seeding and seedling propagation techniques; ② Develop manual dexterity and hand-eye coordination; ③ Experience life’s growth.
Kokedama crafting (KC)	① PPT presentation on common fern growth, kokedama appreciation, and maintenance; ② mixing soil, kneading soil balls, laying moss, tying ropes, and watering	*Asparagus setaceus* seedlings (7.8″-9.8″ height), coarse peat moss, peat soil, spray bottle, jute twine, tweezers, tray, scissors	① Acquiring knowledge of kokedama; ② Mastering kokedama production techniques; ③ Experiencing the zen of kokedama
Flower arranging (FA)	① PPT presentation on flower arrangement knowledge and work appreciation; ② flower mud wrapping, branch trimming, material arrangement	Floral foam, cellophane, floral shears, floral materials, decorative bags	① Learn basic flower arranging techniques; ② Develop esthetic, observational, and spatial perception skills.
Pressed flower card making (PFCM)	① Techniques for making pressed flower greeting cards (card layout design, tweezers handling, glue application)	pressed flower materials, blank cards, tweezers, soft glue, markers	① Practice fine motor skills, esthetics, and patience; ② Be grateful
Decorative bottle painting with dried flowers (DBP)	① Which plants produce dried plant materials? ② Create bottle art freely using selected dried flower materials (bow-tying at the bottle neck)	Dried plant branches and fruits (cotton, lotus pods with seeds, pine cones, etc.), jute twine, acrylic paint and brushes, glass bottle	① Experience textures of dried plant materials; ② Develop imagination, esthetics, and painting skills.

### 2.4 Experimental procedure

Four researchers conducted the experiment. One taught the course, while the others interpreted questionnaires; wore, adjusted, and removed physiological devices; maintained order; guided students after each session; and coordinated with the parents. Only the course instructor and one researcher who monitored the devices remained on-site after the experiment began.

**Figure 1:**
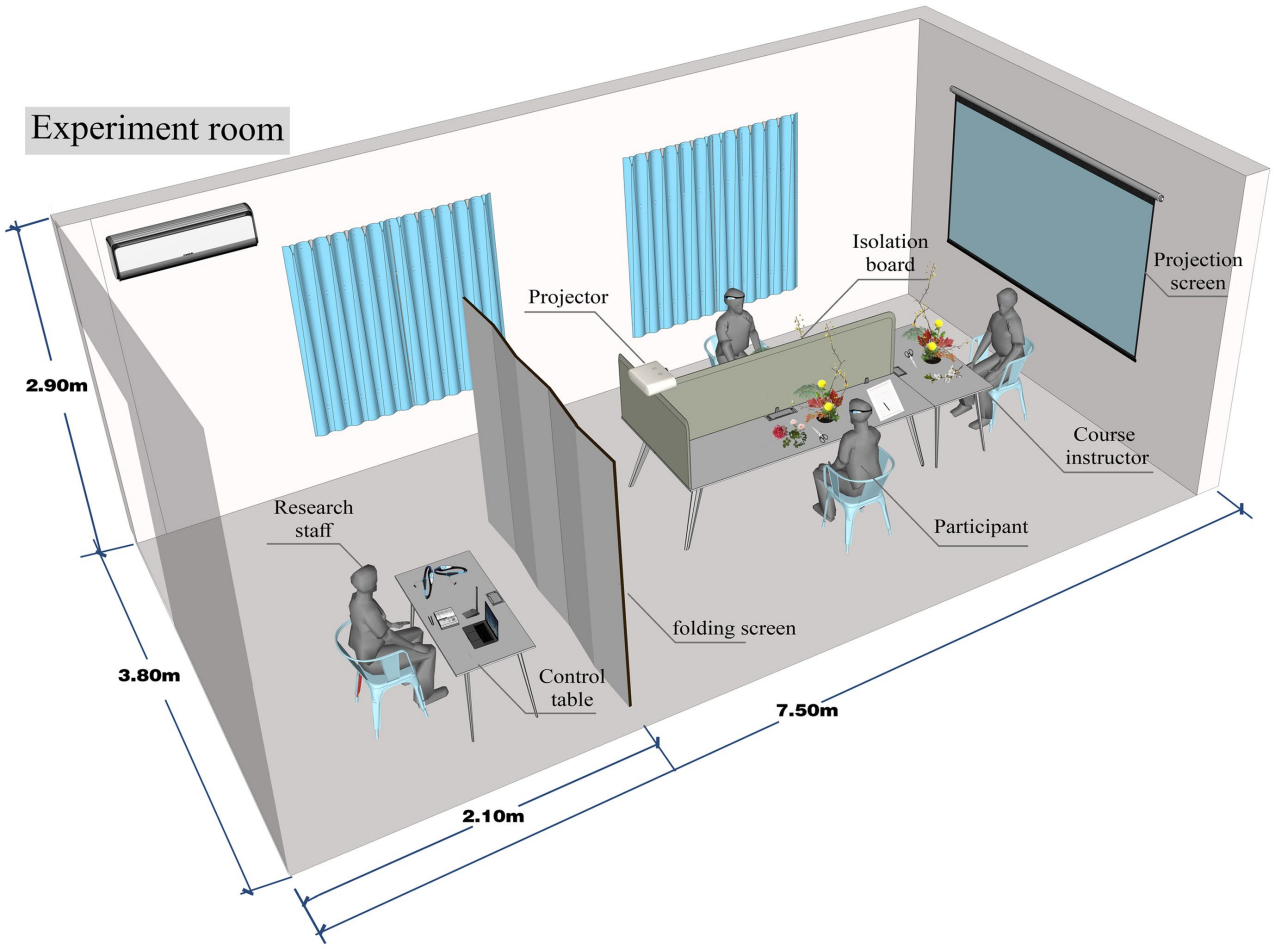
Schematic diagram of experimental setting.

In the experiment, two students faced each other, with the instructor 1.10 meters to one side. After the daily horticultural knowledge course, we set up a partition that separated the two students and the instructor to prevent interference. The researcher monitoring the devices behind the partition (Figure 1).

**Figure 2:**
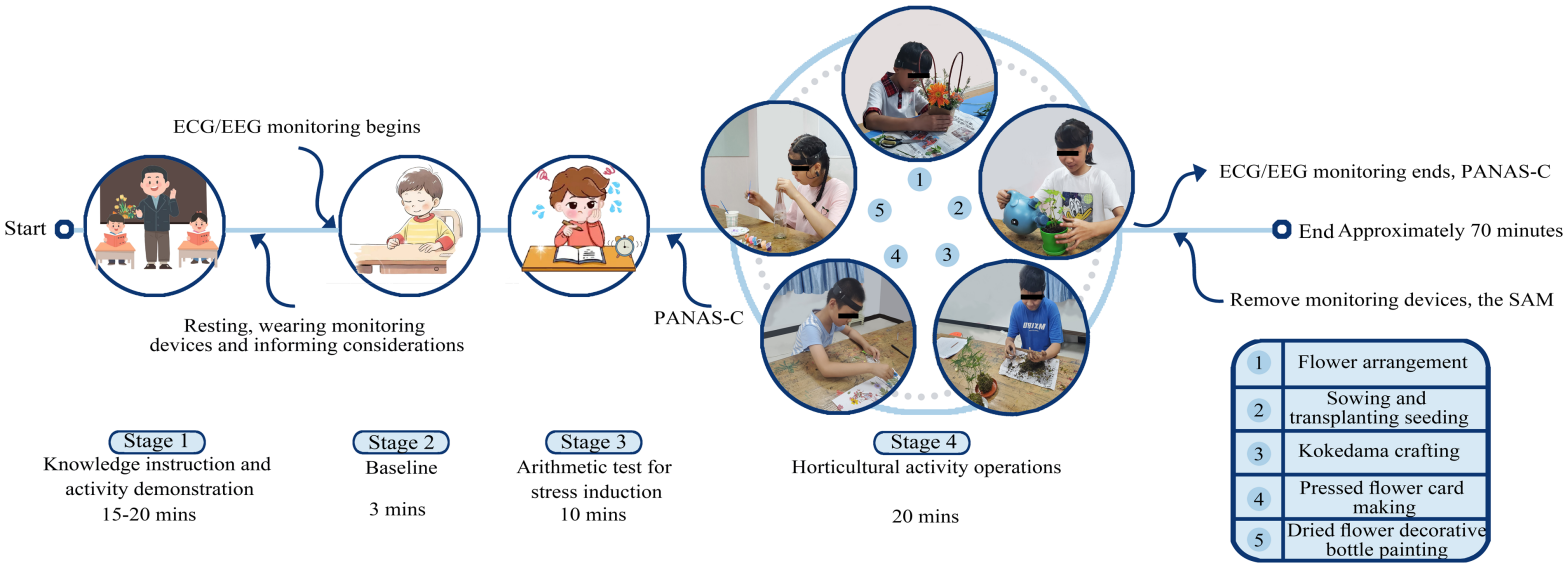
Flowchart of the experimental process.

The experiment proceeded in four stages (Figure 2). The instructor started the day by teaching horticultural knowledge and giving activity demonstrations (15–20 min). Students took a brief break after the course when two research assistants helped them put on physiological monitoring devices, made necessary adjustments, and informed them of essential considerations. Students then underwent three-minute baseline monitoring and were given 10 min to complete an arithmetic test during the stress induction stages, during which a stopwatch timer was set for the final 3 min of the test to increase stress. The students completed the Positive and Negative Affect Schedule for Children (PANAS-C) immediately after stress induction. A 20-min activity operation stage followed, and students performed independently without mutual communication. After the activity, the research staff removed the physiological monitoring devices, and students completed the PANAS-C again, as well as the Self-Assessment Manikin (SAM) questionnaire, based on their experiences. After a total of approximately 70 min, students received horticultural work as a souvenir and left the classroom. The students’ basic information (age, grade, sex, only child status, prior horticultural activity experience) and informed consent forms were completed before the initial activity began.

### 2.5 Instruments and measures

#### 2.5.1 Physiological measures

Stress monitoring physiological indicators included electrocardiogram (ECG) and electroencephalography (EEG) data. The ECG data was recorded in real time using Polar V800 and Polar H10 chest straps (Polar Electro Oy., Kempele, Finland). To process the raw data, we used Kubios HRV Scientific (version 3.5). Artifacts in the inter-beat interval time series of the raw data were corrected automatically, and spectral transformation was conducted using an autoregressive method (Tarvainen et al., 2021).

The heart rate (HR) rises during stress or physiological arousal, and heart rate variability (HRV) indicates changes in the autonomic nervous system. Stress decreases vagal heart regulation and increases sympathetic activity. To measure sympathetic nervous system (SNS) activity, we used Baevsky’s stress index (SI). Long-term HRV analysis (H > 24 h) usually uses time-domain analysis; however, RMSSD and pNN50 can be used for short-term electrocardiogram signal analysis. Thus given its superior statistical properties, reproducibility, and stability, we used RMSSD as the indicator in the vagal nervous system activity time-domain analysis (Lu, 2006). Additionally, sympathetic modulation was indicated by the low-frequency/high-frequency (LF/HF) frequency-domain analysis indicator (0.04–0.15 Hz).

Students’ EEGs were recorded using a wireless portable NeuroSky MindWave-EEG headset (Beijing Oriental Creation Technology Co., Ltd., China). We used α (8–13 HZ) and β (14–30 HZ) EEG waves as stress feedback indicators; the α-EEG (8–13 HZ) indicated calmness and physiological relaxation (Chang and Chen, 2005), while the β-EEG (14–30 HZ) indicated alertness and effortful thinking (Qi et al., 2022). Physical workload or stress can cause α-EEG waves to decrease and β-EEG waves to increase significantly (Hassan et al., 2019a).

#### 2.5.2 Psychological measures

We used two self-reporting questionnaires, the PANAS-C and SAM, to evaluate psychological changes associated with stress and activity. Both questionnaires have been widely employed in the Chinese context and setting (Liu, 2005; Pan et al., 2015; Shu and Ma, 2020; Liu et al., 2021). The PANAS-C has positive and negative dimensions, each with 15 words that describe emotions, which are scored on a five-point scale (1 = *very slightly* or *not at all* to 5 = *extremely*) (Laurent et al., 1999). Cronbach’s α coefficients for the positive and negative subscales were 0.83 and 0.87, respectively, indicating good reliability. We changed the order of the items daily to avoid the inertia effect on students’ choices. The SAM is a nine-point picture-based assessment tool that depicts diverse psychological and emotional states in three dimensions (Bradley and Lang, 1994). The dimensions of “pleasure” (where “1” on the far left represents extremely unpleasant feelings, while “9” represents the highest pleasure on the far right), “arousal” (where “1” represents a state of drowsiness, calmness, and relaxation on the far left, while “9” represents bursting with extreme nervousness and restless on the far right), and “dominance” (where “1” on the left-hand side indicates insufficient confidence and poorly done work, while “9” on the right-hand side indicates excellent confidence levels and well-done work) measure joy, emotional activation, and self-awareness and performance when influenced by external stimuli, respectively. To facilitate students’ understanding, the researchers advised them to visualize themselves as the characters in the picture and mark the interpretation at both ends of the dimensions.

### 2.6 Data analysis

SPSS 26.0 software was used for statistical analysis.

ECG and EEG indicators were set as dependent factors, while five horticulture activities and the reference activity were the independent variables. We used a one-way ANOVA to test baseline-dependent variable differences between activities and conducted paired sample t-tests to examine the effectiveness of stress induction. We compared physiological (Question 1) and affective (Question 2) changes among the students between the stress induction and activity stages using paired sample *t*-tests. To account for individual differences, we converted raw physiological data into percentage change values, as follows:


$$Z{\left(\%\right)}_{i1,i2\ldots i6}=\frac{X_{i1,i2\ldots i6}-Y_{i1,i2\ldots i6}}{Y_{i1,i2\ldots i6}}\times 100$$

where Z = relative change (recovery) values (%); X = mean values during the stress induction stage; Y = Raw values during the activity stage; i1 = HR; i2 = RMSSD; i3 = SI; i4 = LF/HF; i5 = α-EEG; and i6 = β-EEG.

Next, we employed a one-way ANOVA to compare the differences in relative recovery from stress among the six activities. Paired t-tests assessed changes in positive and negative affects before and after activities. The Shapiro–Wilk test showed a non-normality of the SAM data, and Kruskal–Wallis analysis was applied (Question 3).

The activity stage was divided into four time phases (T1–T4) at five-minute intervals. A “model-based” corrected “robust estimator” was chosen as the covariance matrix to ensure the robustness of the model. After testing the model’s goodness of fit with different working correlation matrices, we selected the “exchangeable” matrix with the smallest QIC. After selecting the robust working correlation matrix, we removed non-significant interaction terms and reconstructed the equation. Eventually, we employed maximum likelihood estimation with the smallest QICC and constructed a six (activities) by four (time phases) generalized estimating equation (GEE) model to examine the main effects of activities and time phases on the relative change in HR and EEG levels (Question 4). Furthermore, we included sex and prior horticultural activity experience as factors, with age as a covariate in the GEE. We then examined the interactive effects of “activities × time phases,” “activities × sex,” “time phases × sex,” “activities × prior horticultural activity experience,” and “time phases × prior horticultural activity experience.”

All tests determined significance with a *p*-value below 0.05.

## 3 Results

### 3.1 Stressor validity

A one-way ANOVA revealed no significant differences in the baseline data between activities for the HR (*p* = 0.426), RMSSD (*p* = 0.320), LF/HF (*p* = 0.099), SI (*p* = 0.533), α-EEG (*p* = 0.458), and β-EEG (*p* = 0.730). Compared with the baseline, the values of HR, LF/HF, SI, and β-EEG increased after the stress induction stage, whereas RMSSD and α-EEG decreased significantly (see Supplementary Table 1). These findings suggested that the arithmetic tests raised the students’ physiological stress levels effectively.

### 3.2 Effects of the activities on physiological indicators

#### 3.2.1 Recovery effects of the activities

As shown in Table 2, the ECG data revealed that mean values for the students’ HR and SI during the activity stages of the horticultural activities were significantly lower than in the stress induction phase, whereas the RMSSD were significantly higher. The frequency domain analysis results were inconsistent. The SATS, PFCM, and DBP had lower LF/HF mean values during the operational stage than in the stress induction stage; however, the FA and KC had the opposite pattern. Regarding EEG, α-EEG was significantly higher during the operational stages compared with the stress induction stage in horticultural activities, whereas β-EEG was significantly lower. Thus, the participants underwent less physiological stress during the horticultural operational stage, with the exception of their LF/HF during the FA and KC activities.

**Table 2:** Comparison of physiological indicators means values before and after the Horticultural activities.

Indicators	Horticultural activities	Stress-induced stage (M ± SD)	Activity-operated stage (M ± SD)	*p*	*t*	Cohen’s *d*
HR (bpm)	FA	102.28 ± 12.68	95.90 ± 10.58	<0.001	6.267	0.546
	SATS	100.85 ± 12.10	94.01 ± 10.99	<0.001	8.880	0.592
	KC	100.50 ± 11.80	95.62 ± 10.98	<0.001	6.727	0.428
	PFCM	104.03 ± 12.37	97.06 ± 10.20	<0.001	9.887	0.615
	DBP	101.66 ± 12.00	95.27 ± 11.11	<0.001	8.627	0.552
RMSSD (ms)	FA	21.53 ± 11.24	24.77 ± 12.34	<0.001	−3.291	−0.274
	SATS	20.07 ± 9.22	24.89 ± 11.87	<0.001	−4.923	−0.452
	KC	20.77 ± 10.16	23.74 ± 11.91	0.002	−3.283	−0.267
	PFCM	18.47 ± 10.52	21.79 ± 10.42	<0.001	−3.991	−0.371
	DBP	20.86 ± 11.81	25.21 ± 15.13	<0.001	−4.621	−0.320
LF/HF	FA	2.26 ± 1.18	2.73 ± 2.01	0.033	−2.202	−0.285
	SATS	2.61 ± 1.47	2.50 ± 1.68	0.582	0.525	0.076
	KC	2.50 ± 2.03	2.54 ± 1.54	0.820	−0.343	−0.022
	PFCM	3.12 ± 2.49	2.97 ± 2.02	0.500	0.566	0.066
	DBP	2.53 ± 1.91	2.39 ± 2.55	0.521	0.584	0.062
SI	FA	19.07 ± 7.83	15.40 ± 4.57	<0.001	4.652	0.574
	SATS	19.10 ± 6.13	15.21 ± 5.37	<0.001	6.397	0.675
	KC	18.82 ± 6.15	16.02 ± 5.71	<0.001	4.307	0.473
	PFCM	20.87 ± 7.71	16.93 ± 5.33	<0.001	4.997	0.594
	DBP	19.61 ± 7.59	16.50 ± 6.03	<0.001	5.816	0.453
α-EEG (Power units)	FA	24433.50 ± 4948.52	30815.75 ± 7139.58	<0.001	−9.034	−1.039
	SATS	23923.38 ± 5132.80	29357.23 ± 5359.23	<0.001	−10.086	−1.036
	KC	22955.81 ± 5749.69	29075.88 ± 7753.31	<0.001	−8.020	−0.897
	PFCM	24680.56 ± 6208.48	30210.42 ± 7267.88	<0.001	−9.897	−0.818
	DBP	23300.65 ± 4760.00	29436.52 ± 5666.85	<0.001	−11.842	−1.172
β-EEG (Power units)	FA	23020.88 ± 6291.99	18054.52 ± 5121.02	<0.001	10.028	0.866
	SATS	21197.06 ± 5960.87	17152.35 ± 4062.01	<0.001	8.108	0.793
	KC	21886.71 ± 6667.41	17479.31 ± 5458.79	<0.001	10.445	0.723
	PFCM	21292.40 ± 4828.76	16726.60 ± 4801.76	<0.001	9.935	0.948
	DBP	21201.52 ± 5065.67	16974.40 ± 4685.41	<0.001	7.922	0.866

#### 3.2.2 Comparison of the relative recovery effects of the activities on stress

**Figure 3:**
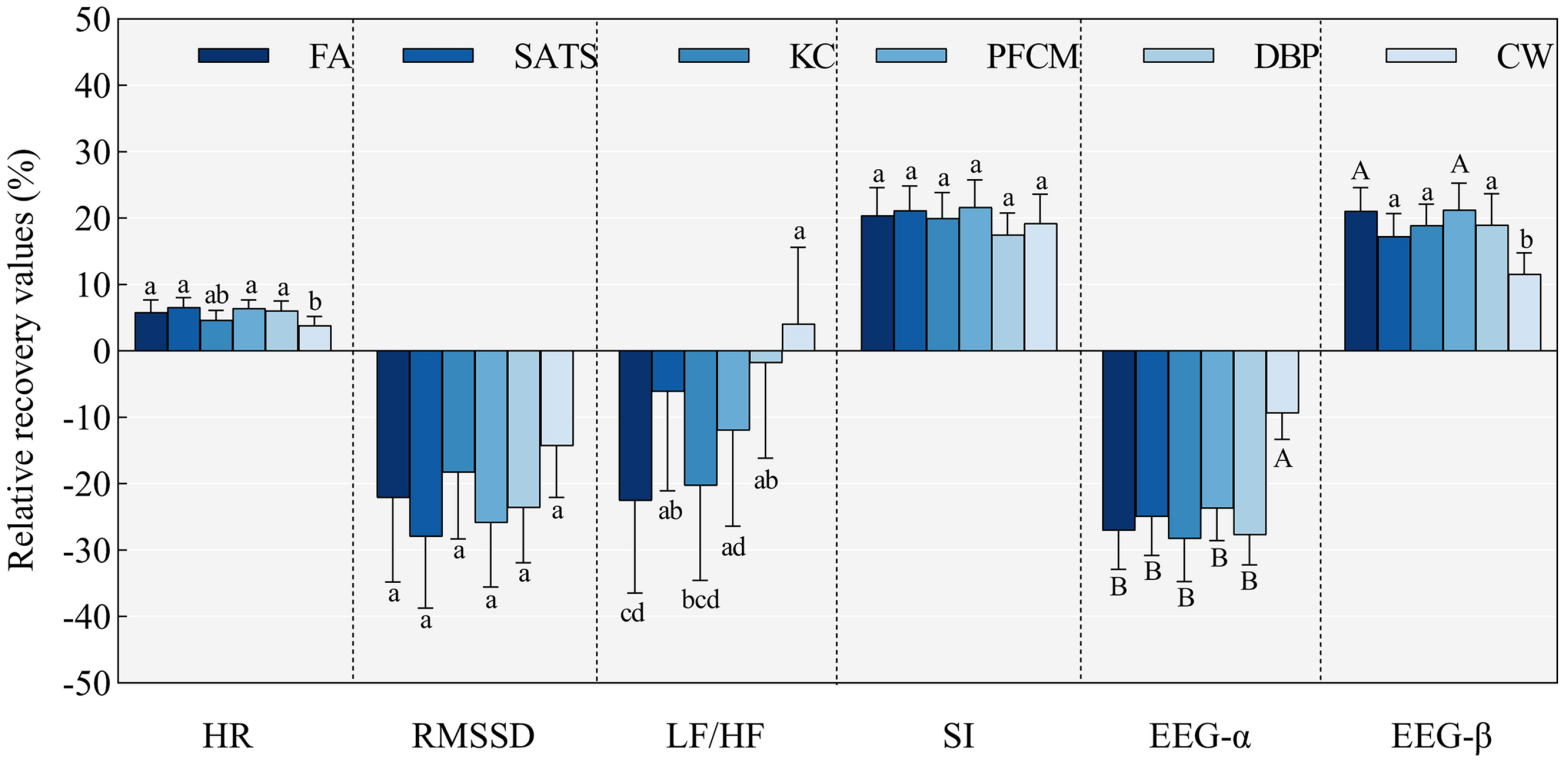
Comparison of recovery values for activities in response to stress.

As shown in Figure 3, the HR, RMSSD, and SI results were relatively consistent, indicating that the horticultural activities elicited better recovery than the comparison activity. However, only the HR showed significant differences between the activities (*F* = 2.327, *p* = 0.043, $\eta_{p}^{2}$ = 0.040). The least significant difference post-hoc test revealed that the FA (*p* = 0.044), SATS (*p* = 0.007), PFCM (*p* = 0.011), and DBP (*p* = 0.024) activities had significantly higher relative recovery values than the reference activity. However, we observed no significant differences among the horticultural activities. Notably, the reference activity exhibited better relative recovery in the LF/HF than in the FA and KC activities. The change values for activities showed significant differences in both α-EEG (*p* < 0.001) and β-EEG (*p* = 0.002). Differences between each horticultural activity and the reference activity were significant, but they were not significant among the horticultural activities.

In sum, the horticultural activities outperformed the reference activity in physiological stress recovery, except for the ECG frequency domain indicators; however, the recovery benefits were not much different between the different horticultural activities. Comparatively, the SATS and FA showed better results according to the ECG and EEG, respectively.

#### 3.2.3 Changes in relative recovery by activity stage

Table 3 presents the results of GEE_S_ constructed based on HR and EEG. Regarding the students’ HR, the main effect of sex was significant: The boys had a relative recovery value of 3.681%, lower than the girls, indicating that the horticultural activities benefitted girls relatively more. The “activities × time phases” interaction demonstrated statistical significance.

**Table 3:** GEEs for relative stress changes in the activity stage.

Factors & Covariates	EEG				ECG	
	α-EEG		β-EEG		HR	
	Wald *χ*^2^	*p*	Wald *χ*^2^	*p*	Wald χ^2^	*p*
Activity	72.674	<0.001	22.038	0.001	16.868	0.005
Time	1.332	0.722	5.164	0.160	61.011	<0.001
Age	1.587	0.208	0.913	0.339	0.419	0.518
Prior horticultural activity experience	5.594	0.018	0.397	0.528	1.706	0.191
Sex	2.258	0.133	2.685	0.101	34.989	<0.001
Activity* Time	31.602	0.007	/	/	66.616	<0.001
Activity* Sex	0.576	0.989	/	/	/	/
Time* Sex	3.735	0.291	/	/	/	/
Activity* Prior horticultural activity experience	13.549	0.019	/	/	/	/
Time* Prior horticultural activity experience	0.919	0.821	/	/	/	/

**Figure 4:**
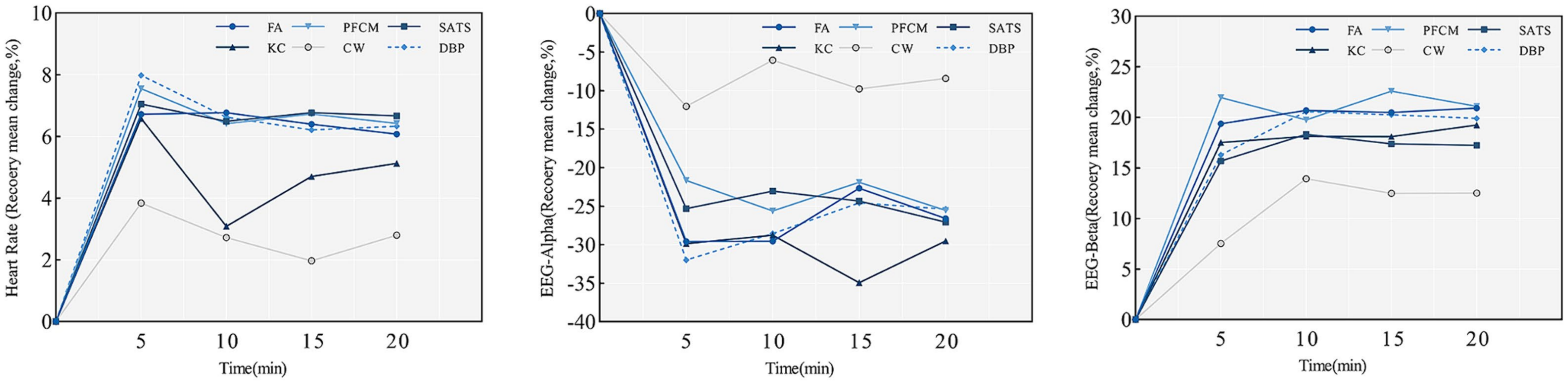
Trends in HR and EEG relative recovery values among elementary students.

The simple effects analysis with time phases yielded the following results. Students released the most stress in T1, during which the horticultural activities, except for the FA activity, offered the best recovery benefits. The PFCM and DBP activities had significantly higher relative recovery values in T1 than in T2, T3, and T4. The relative change values of the FA activity increased in T2, whereas those of the other horticultural activities decreased. Further, the KC values were significantly lower in T2 than in T1, T3, and T4. The FA values gradually declined from T3 to T4, whereas the KC values gradually recovered. In contrast to the DBP activity, the PFCM values initially increased and then decreased in relative recovery levels. However, these changes were not significant. Generally, we found no overarching rule governing the fluctuations in relative change values across the horticultural activities (Figure 4). The simple effects of activities showed that except for the KC activity in T2 and T4, the horticultural activities in the remaining time phases had significantly higher relative recovery values than the CW (*p* = 0.001). The FA had significantly higher relative recovery values in T2 than the KC (*p* = 0.006). Horticultural activities in other time phases had similar relative recovery effects. From a cardiovascular system perspective, the effects on elementary students’ stress recovery varied with the content of the activity.

Regarding EEG, “activities × time phases” and “activities × prior horticultural activity experience” had a significant interaction effect on the α-EEG, and “activities” had a significant main effect on the β-EEG. Horticultural activities had different relative change trends over time (Figure 3). The “time” simple effect showed that, in T3, the relative change values for the FA activity were significantly less than those at T1 (*p* = 0.017) and T2 (*p* = 0.002), whereas the other horticultural activities showed similar recovery values across the time phases. However, the KC activity showed a trend of improved recovery in T2–T3 that aligned with its trends in HR. The “activity” simple effect showed that the KC activity offered higher recovery compared with the other horticultural activities in T3. There were no significant differences among horticultural activities in the other phases, although performed better than the reference activity.

**Figure 5:**
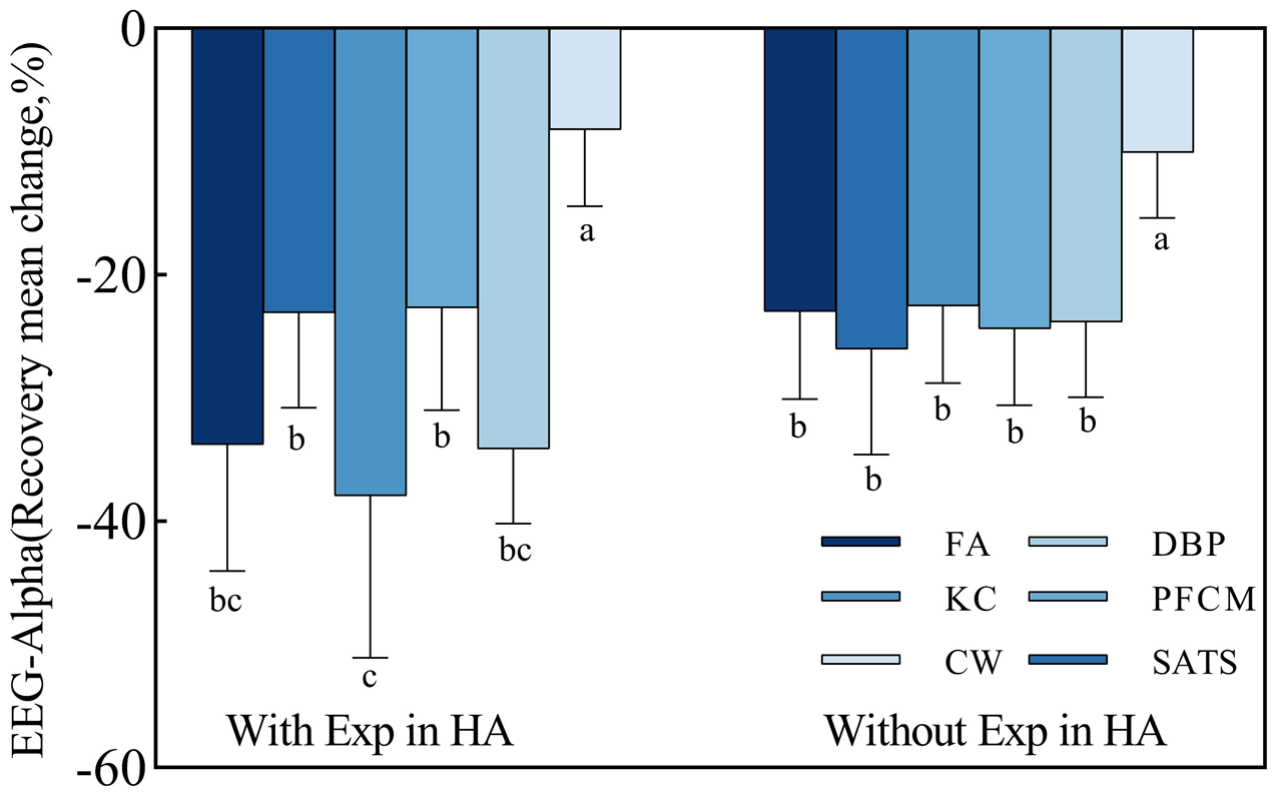
Interaction effect of “Activities” and “Prior horticultural activity experience or not” on α-EEG relative recovery values. FA, Flower Arrangement; SATS, Sowing And Transplanting Seeding; KC, Kokedama Crafting; PFCM, Pressed Flower Card Making; DBP, Decorative Bottle Painting; CW, Composition Writing; Error bars, 95%CI; *N* = 48.

Figure 5 displays the results of a simple effect analysis of “activities × prior horticultural activity experience.” Horticultural activities significantly improved α-EEG recovery compared with the CW, regardless of whether students had prior horticultural activity experience. The recovery effect of the KC activity was superior to that of the SATS and PFCM activities for students with horticultural experience. Students without experience recovered similarly between different horticultural activities. The recovery levels of β-EEG in horticultural activities varied over time without a consistent pattern (Figure 4). The main effect of the activities indicated that the horticultural activities offered better recovery than the CW. The EEG results corresponded with the HR, providing solid evidence from a physiological perspective that the stress-relieving benefits of the horticultural activities for elementary students were distinct from those of the reference activity.

### 3.3 Effects of the activities on psychological indicators

#### 3.3.1 Positive and negative affects

The results of the one-way ANOVA showed no significant difference in positive (*p* = 0.667) or negative (*p* = 0.874) emotional levels before the activity. Post-test, the positive levels increased, and the negative levels decreased (Table 4). Improvements in positive (*F* = 19.625, *p* < 0.001, $\eta_{p}^{2}$ = 0.258) and negative (*F* = 25.721, *p* < 0.001, $\eta_{p}^{2}$ = 0.313) affect differed significantly across activities. Horticultural activities significantly increased the positive affect compared with the reference activity (*p* < 0.001), with the most significant response in the FA activity (from 24.13 ± 5.90 to 50.25 ± 8.79). Thus, the FA was the best activity for improving students’ positive affect. Compared with the reference activity, horticultural activities significantly decreased negative affect (*p* < 0.001), although the differences were not significant. The results imply that the elementary students’ positive affect was significantly affected by the type of horticultural activities they participated in and that horticultural activities led to a more marked improvement in students’ affect than the reference activity.

**Table 4:** Effect of activities on PANAS scores.

Psychological indicators	Activity	Stress-induced phase (M ± SD)	Activity-operated phase (M ± SD)	*t*	*p*	Cohen’s *d*
PA	FA	24.13 ± 5.90	50.25 ± 8.79	−21.473	<0.001	−3.489
	SATS	22.67 ± 6.35	44.52 ± 12.95	−12.126	<0.001	−2.142
	KC	22.40 ± 5.20	45.10 ± 11.47	−14.771	<0.001	−2.549
	PFCM	22.92 ± 5.32	42.27 ± 11.96	−12.935	<0.001	−2.090
	DBP	22.85 ± 5.50	41.83 ± 11.96	−11.100	<0.001	−2.039
	CW	22.42 ± 4.70	26.40 ± 6.34	−5.224	<0.001	−0.713
NA	FA	32.35 ± 9.56	17.40 ± 3.33	12.210	<0.001	2.088
	SATS	31.85 ± 10.34	16.60 ± 2.03	10.700	0.001	2.036
	KC	30.85 ± 8.97	16.75 ± 2.99	11.605	<0.001	2.108
	PFCM	32.44 ± 9.05	16.79 ± 3.48	11.868	<0.001	2.282
	DBP	32.96 ± 8.33	17.42 ± 3.41	13.178	<0.001	2.442
	CW	31.31 ± 7.18	26.83 ± 4.83	3.703	<0.001	0.732

FA, Flower Arrangement; SATS, Sowing And Transplanting Seeding; KC, Kokedama Crafting; PFCM, Pressed Flower Card Making; DBP, Decorative Bottle Painting; HR, Heart Rate; RMSSD, Root Mean Square of Successive Differences; LF/HF, Low Frequency Power/High Frequency Power; SI, Stress Index; PA, Positive Affect; NA, Negative Affect; M ± SD, Mean ± Standard Deviation; The effect size is reported by Cohen’s d.

#### 3.3.2 Self-Assessment Manikin

**Figure 6:**
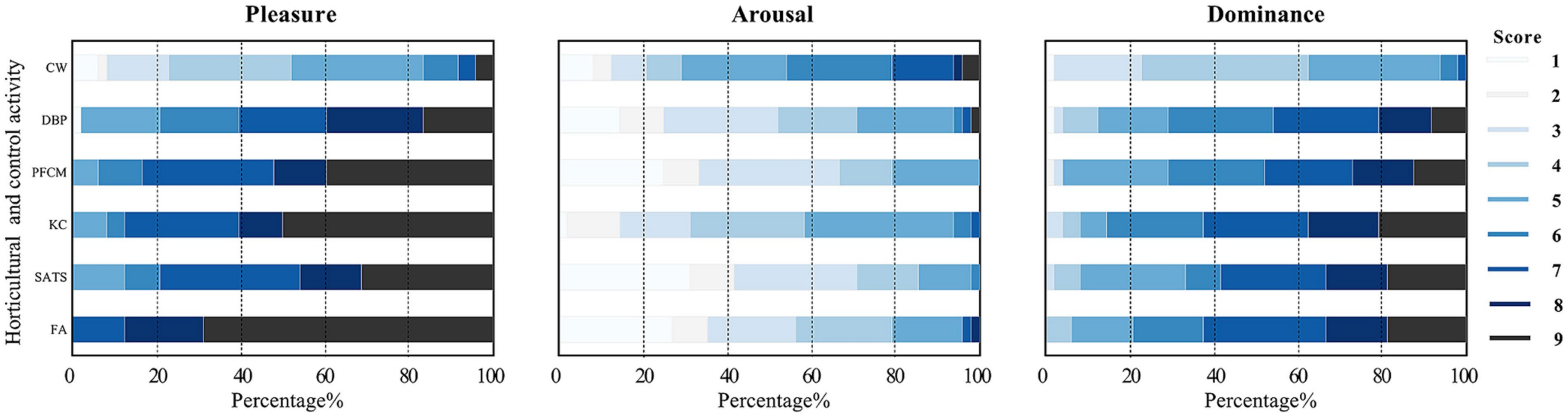
Students’ Self-Assessment Manikin scale evaluation on activities.

The Kruskal–Wallis test revealed significant differences in the distribution of scores for pleasure, arousal, and dominance among the activities (see Supplementary Table 2). For pleasure, the median scores for the FA activity were the highest, approaching 9 points (Figure 6). The FA and KC were the most preferred horticultural activities, followed by the PFCM, whereas the SATS and DBP were the least preferred. Regarding arousal, students were typically calm during activities. Except for the KC, the horticultural activities differed significantly from the CW in score distribution. This implied that the students experienced some fluctuations in their emotions when participating in KC, consistent with the T2 phase’s lower stress recovery in terms of HR. Pairwise comparisons showed significant differences between the horticultural activities and the reference activity (adj. *p <* 0.001), but not among the horticultural activities, for the dominance dimension. These results indicated that the horticultural activities boosted students’ self-confidence more than the comparison activity, giving them a greater sense of accomplishment, even while undertaking horticultural activities that they found slightly difficult.

The results of the Mann–Whitney U test showed that boys and girls did not differ significantly in the pleasure (*Z* = −0.475, *p* = 0.634), arousal (*Z* = −1.084, *p* = 0.279), or dominance dimensions (*Z* = −0.060, *p* = 0.952). The score distribution charts showed that the FA activity made both boys and girls happy; however, the boys thought they were better at the KC activity, whereas for the girls, the FA and SATS activities calmed them the most (see Supplementary Figure 1).

## 4 Discussion

### 4.1 Physiological stress recovery effect of horticultural activities

The HR, RMSSD, SI, and EEG results indicated that 20 min of horticultural-related activities could significantly reduce physiological stress in elementary students. Previous research has also examined the correlation between engagement in horticultural activities and individuals’ physiological stress levels, employing various indicators as measures. Hassan et al. (2018) reported significant decreases in older women’s blood pressure after a 15-min flower planting activity compared with a control group. Another study that used flower appreciation and farming work reported decreased salivary cortisol levels and increased oxytocin and parasympathetic nervous system activity in older adults (Watanabe et al., 2021). The results of our study not only align with previous work focused on older populations but also provide evidence for a younger cohort. Additionally, these results are similar to those from research on stress recovery through various forms of contact with nature (Abd-Alhamid et al., 2020; Yin et al., 2020). Horticultural-related activities serve as a medium for connection with nature, which can help lower physiological stress in elementary students and enable their transition from tension anxiety to a state of calmness and relaxation. An evolutionary theory position may explain these effects: Humans are subconsciously drawn to nature because savannahs and environments where water was present helped them survive (Kaplan and Talbot, 1983). The type of non-taxing attention paid to nature and plants is a key mechanism for people to recover from mental fatigue. This study’s results also support the psycho-evolutionary framework that holds that exposure to non-threatening nature after a stressor leads to more positive emotions and lower physiological arousal (Ulrich et al., 1991). Furthermore, gardening could achieve training effects similar to other activities but with less perceived physiological stress (Tao et al., 2022).

We also found lower physiological stress during the CW activity than in the pre-test. The reason for this result might be that the stress induction had a practical effect, and using a timer more vividly replicated the real-life scenario at school. Notably, although the CW was a learning activity, it was not a test. Huang et al. (2020) demonstrated that even in environments without vegetation, moderate viewing duration and relatively rich scenes have a therapeutic effect.

Increased LF/HF indicates a stressed state. We noted an inconsistency in the LF/HF for the FA and KC activities compared with other ECG indicators, implying that the horticultural activities both improved and exacerbated stress among the students. Differences have also emerged in previous research, possibly owing to horticultural activities triggering more complex physiological responses (Li et al., 2020). Another possible explanation relates to the intricate balance control mechanism of the autonomic nervous system—systematic errors may account for the aforementioned result. Thus, physiological indicators require a multi-angle assessment, and subjective reports should be combined to comprehensively evaluate the recovery effects provided by different activities.

### 4.2 Differential relative recovery for different activity types

Although the five horticultural activities significantly relieved stress, we found no significant difference in their recovery potential across indicators. Similar results have been found by other studies examining the benefits of different types of horticultural activity for patients with dementia and children with maladjustment issues (Gigliotti et al., 2004; Lee et al., 2018). One possible explanation is the intricate nature of physiological intrinsic feedback. The difficulty levels, metabolic equivalents, and research environments among horticultural activities are similar. Consequently, the effects of activities on physiological indicators may not differ significantly in terms of sympathetic or central nervous system regulation. The relatively short intervention duration may also be a factor. Nonetheless, we noted the following three points. First, horticultural-related activities yielded better recovery than the reference activity. Certain activities recorded significant differences in HR indicators. Thus, the horticultural activities had inherent characteristics that may make them more effective in alleviating students’ stress. Second, previous studies have suggested that KC relaxed older adults more than SATS (Tu et al., 2020). As such, the stress-relief benefits of different horticultural activities cannot be generalized across age populations, given the variations in the difficulty of these activities and the traits of the participants. The indicator trends in phases T2–T3 (Figure 3) were related to activities that involved planting seedlings in a soil ball, twining, and fastening twine, which required hand coordination and may have activated the students’ sympathetic nervous system, complicating their stress recovery. In China, theory is prioritized over practice for contemporary elementary students, weakening their hands-on skills (Qiao and Zhou, 2020). Meanwhile, the operation of SATS is relatively simple. Third, the EEG results showed that the FA activity had the best relative recovery effect, which was inconsistent with the ECG results. This discrepancy could be attributed to the fact that ECG and EEG data reflect feedback from sympathetic nerves in the cardiovascular system and central nerves in the cortex, respectively. The α-EEG indicates occipital and parietal cortex activity involved in visual processing (Hsu and Wang, 2013; Rehman and Al Khalili, 2022), and the bright, pastel colors of the FA activity materials may have boosted children’s brain waves by enhancing their visual perception.

### 4.3 Trends and interactions in physiological indicators at different activity stages

The horticultural activities were related to significantly improved relative recovery among the students compared with the reference activity during all time phases except for the HR in the T2 and T4 phases of the KC activity. Thus, the advantages of horticultural activities for stress relief may not have been limited to one or a few time phases, but rather all stages of the activities.

Trends in stress recovery were inconsistent across the horticultural activities, although the initial 5–10 min of these activities exhibited superior recovery benefits compared with the later phases. As we did not focus on the mechanisms of stress recovery, we could not confirm whether this may be due to the inherent factors of the activity (e.g., complexity and difficulty of steps) eliciting more intricate physiological changes and inducing confounding effects on the recovery outcome in the later stage. A single horticultural activity for children typically lasts 60 to 120 min (Park et al., 2016), with little evidence linking the duration to recovery efficiency. Longer or shorter experiments need to be conducted to identify the optimal duration of activity for restoration.

We also found that girls recovered 3.68% more than boys in terms of their HR. Although we could not assess the underlying mechanism of this result, we could assume the role of biological and social differences between girls and boys in their varied stress responses (Jiang et al., 2014). When stressed by grades or performance, men may feel more pressure than women (Jin et al., 2023). Thus, horticultural activities may provide girls with a greater recovery effect in the same time frame as boys.

In the GEE for α-EEG, the interaction between prior horticultural experience and activities was significant, indicating that horticultural experience potentially impacted the stress recovery of elementary students. For students lacking horticultural experience, the FA, KC, and DBP activities demonstrated higher effectiveness compared with SATS and PFCM activities. Our findings confirmed that elementary students recover better when interacting with actual plants and materials that resemble nature. As previously reported, “fascination” in a restorative environment is a crucial element that elicits involuntary attention (Kaplan and Kaplan, 1989). The delicate colors of the FA materials and the carefree posture of *Asparagus setaceus* seedlings, which closely resemble the layered structure of natural vegetation, undoubtedly served as engrossing objects for the children. This attraction may have been even stronger for children without prior experience with similar activities, providing a plausible explanation for the aforementioned difference. Through patterns of “living plant crafts” (e.g., the KC activity), indirect contact with nature is made interesting. People typically achieve closeness to nature by playing domestication roles (e.g., gardening and caring for pets), thereby enhancing their “compatibility” with nature (Kaplan, 1995).

### 4.4 Elementary students’ horticultural activity preferences

Elementary students found the FA and KC activities more effective in improving their positive emotions and providing greater happiness and enjoyment than the other horticultural activities. The petals of the *Eustoma russellianum*, *Dendranthema morifolium* cv. “pompon,” and *Rosa* “Barbie Bubbles” have gentle hues (cool and intermediate) and textures, whereas the *Eucalyptus pulverulenta* “Baby Blue” has an invigorating aroma that further enhances visual, tactile, and olfactory sensations. These elements may create a cozier, more serene atmosphere and promote a subliminally calming effect in children (Koga and Iwasaki, 2013; Neale et al., 2021), while also evoking positive emotions (Xiao et al., 2021). Meanwhile, students also favored horticultural activities that incorporated live plants, indirectly supporting Wilson’s biological and genetic viewpoint that “humans possess a fundamental, genetically-based desire and inclination toward living and lifelike entities, even with minimal connection to nature” (Wilson, 1986). Playing with mud comes naturally to most children, and this might be why students considered molding soil balls into moss balls intriguing. Dirt, water, fallen leaves, and other materials in the natural world offer children valuable opportunities to play and learn, encouraging them to explore, think critically, and seek out more engaging activities (Nicholson, 1972). This also indicates that elementary students’ emotional and affective recovery varied depending on the content and materials of the horticultural activities.

### 4.5 Limitations

While our study confirmed the positive effects of horticultural-related activities on the psychophysiological stress of elementary students and the differences in recovery benefits across different activities, some limitations warrant further research. First, although the sample size met the estimates of G*Power, a larger and more diverse sample, including a wider age range, is necessary for more comprehensive conclusions. Second, the gender ratio is close to 1:1, which may affect the research sample’s representativeness and the results’ reliability and generalizability. Therefore, there is a need to control for gender proportions. Third, we did not fully consider demographic factors. Family environments and backgrounds (e.g., parents’ income, single-parent households, and intergenerational care) may also affect the dependent variables, as children’s preferences often mirror those of their parents (Meidenbauer et al., 2019). Finally, all study participants were from urban regions. However, rural children may exhibit differing cognition, stress tolerance, and health behaviors because of their natural growth environment (Hope and Bierman, 1998).

## 5 Conclusion

Engaging in horticultural-related activities, which can serve as an interactive experience with natural elements and provide entertainment, could effectively mitigate stress and fatigue levels among elementary students. After participating in horticultural activities, the students in this study had significantly decreased physiological stress and reported a more positive emotional experience. Horticultural-related activities exhibited noticeably greater benefits for stress recovery than the reference activity of freewriting. Although students’ physiological stress levels did not differ greatly among different horticultural activities in certain measures, the recovery effect was enhanced by the use of soft-colored and vibrant live plant materials, such as in the FA and SATS activities. We also noted the importance of the fun factor. The KC activity, which represented the “living plant crafts” activity type and involved slightly complex steps, was preferred by students despite offering low physiological stress recovery. Conversely, the SATS activity, with simpler steps and relatively monotonous content, required less physiological strain from students, but was not rated as the most engaging. Thus, interest and activity difficulty must be considered when planning elementary school-level horticultural programs. The activity type of art or pressed flower crafts, exemplified by the PFCM activity, and other artistic activities, represented by the DBP activity, also exhibited effective stress reduction in psychophysiological measures. Nevertheless, their efficacy in fostering positive affect was significantly lower than the other three types of horticultural activity.

It should be noted that our study primarily focused on the benefits of active indoor horticultural activities for elementary students, particularly in terms of stress relief. Further studies are needed to explore the benefits of outdoor or passive horticultural activities, such as hiking and scenic viewing, and to ascertain how they compare to indoor horticultural activities. It is also beneficial to investigate the healing benefits of horticultural-related courses for elementary students who may experience anxiety and depression. These research gaps require prompt attention in future studies.

Horticultural activities can reduce stress and improve the emotional well-being of elementary students while contributing to their moral, intellectual, artistic, and labor education. We expect our findings to guide educational authorities in selecting and organizing horticultural activity programs for elementary students.

## Data availability statement

The original contributions presented in the study are included in the article/Supplementary material, further inquiries can be directed to the corresponding author.

## Ethics statement

The studies involving humans were approved by the Ethics Committee of College of Landscape Architecture and Arts, Northwest A&F University. The studies were conducted in accordance with the local legislation and institutional requirements. Written informed consent for participation in this study was provided by the participants’ legal guardians/next of kin. Written informed consent was obtained from the individual(s), and minor(s)’ legal guardian/next of kin, for the publication of any potentially identifiable images or data included in this article.

## Author contributions

LG: Writing – original draft, Visualization, Software, Methodology, Investigation, Formal analysis, Data curation, Conceptualization. WX: Writing – review & editing, Validation, Software, Project administration, Methodology, Investigation, Data curation. YS: Writing – review & editing, Validation, Project administration, Methodology, Investigation, Data curation. SG: Writing – review & editing, Validation, Project administration, Methodology, Data curation. CX: Writing – review & editing, Validation, Project administration, Methodology, Data curation. XZ: Writing – review & editing, Methodology. XL: Writing – review & editing. QZ: Writing – review & editing. YZ: Writing – review & editing, Validation, Supervision, Conceptualization.
