# Supplementary material for: Which horticultural activities are more effective for children’s recovery from stress and mental fatigue? A quasi-experimental study
Source: Front Psychol. 2024 Apr 12;15:1352186. doi: 10.3389/fpsyg.2024.1352186 (PMC11050040; doi:10.3389/fpsyg.2024.1352186)
Supplement: Supplementary file 1 [file Table_1.DOCX]

Supplementary Material

**Supplementary Table 1.** physiological level changes between the baseline and stress induction phases.

| Indicators | Phase | M±SD | Difference  (95%CI) | Paired t-test | | |
| --- | --- | --- | --- | --- | --- | --- |
|  |  |  |  | *t* | *P* | Cohen’s *d* |
| HR  (bpm) | baseline | 93.95±11.85 | -7.86  (-8.56,-7.16) | -22.10 | 0.000 | 0.66 |
|  | stress | 101.80±12.02 |  |  |  |  |
| RMSSD  (ms) | baseline | 28.75±15.86 | 8.41  (4.45,12.37) | 4.18 | 0.000 | 0.32 |
|  | stress | 20.34±10.86 |  |  |  |  |
| LF/HF | baseline | 2.04±2.49 | -0.64  (-0.89,-0.40) | -5.15 | 0.000 | 0.29 |
|  | stress | 2.68±1.93 |  |  |  |  |
| SI | baseline | 17.24±6.77 | -2.31  (-2.89,-1.74) | -7.86 | 0.000 | 0.33 |
|  | stress | 19.56±7.11 |  |  |  |  |
| EEG-α  （Power units） | baseline | 32282.26±8368.45 | 8200.12  (7405.80,8994.50) | 20.32 | 0.000 | 1.13 |
|  | stress | 24082.11±5917.17 |  |  |  |  |
| EEG-β  （Power units） | baseline | 14371.01±4523.55 | -6954.10  (-7579.81,-6328.39) | -21.88 | 0.000 | 1.33 |
|  | stress | 21325.11±5781.84 |  |  |  |  |

HR=Heart Rate, RMSSD= Root Mean Square of Successive Differences, LF/HF=Low Frequency Power/High Frequency Power, SI=Stress Index, PA=Positive Affect, NA=Negative Affect, M±SD=Mean ± Standard Deviation, 95%CI=95%Confidence Interval, The effect size is reported by Cohen’s d.

**Supplementary Table 2.** Students' self-evaluation of emotions in activities.

| Activity | Pleasure | | Arousal | | Dominance | |
| --- | --- | --- | --- | --- | --- | --- |
|  | M  (SD) | Median  (P25,P75) | M  (SD) | Median  (P25,P75) | M  (SD) | Median  (P25,P75) |
| FA | 8.56  (0.17) | 9.00  (8.00,9.00) | 3.13  (1.72) | 3.00  (1.00,4.00) | 6.88  (1.49) | 7.00  (6.00,8.00) |
| SATS | 7.44  (1.35) | 7.00  (7.00,9.00) | 2.73  (1.46) | 3.00  (1.00,4.00) | 6.67  (1.68) | 7.00  (5.00,8.00) |
| KC | 7.90  (1.30) | 8.50  (7.00,9.00) | 4.02  (1.26) | 4.00  (3.00,5.00) | 6.94  (1.60) | 7.00  (6.00,8.00) |
| PFCM | 7.69  (1.27) | 8.00  (7.00,9.00) | 2.96  (1.44) | 3.00  (1.25,4.00) | 6.48  (1.58) | 6.00  (5.00,8.00) |
| DBP | 6.88  (1.61) | 7.00  (6.00,8.00) | 3.52  (1.68) | 3.00  (2.25,5.00) | 6.25  (1.64) | 6.00  (5.00,7.00) |
| CW | 4.44  (1.64) | 4.00  (4.00,5.00) | 5.06  (1.97) | 5.00  (4.00,6.00) | 4.19  (1.02) | 4.00  (4.00,5.00) |
| H | 120.518 | | 54.082 | | 81.810 | |
| *P* | ＜0.001 | | ＜0.001 | | ＜0.001 | |


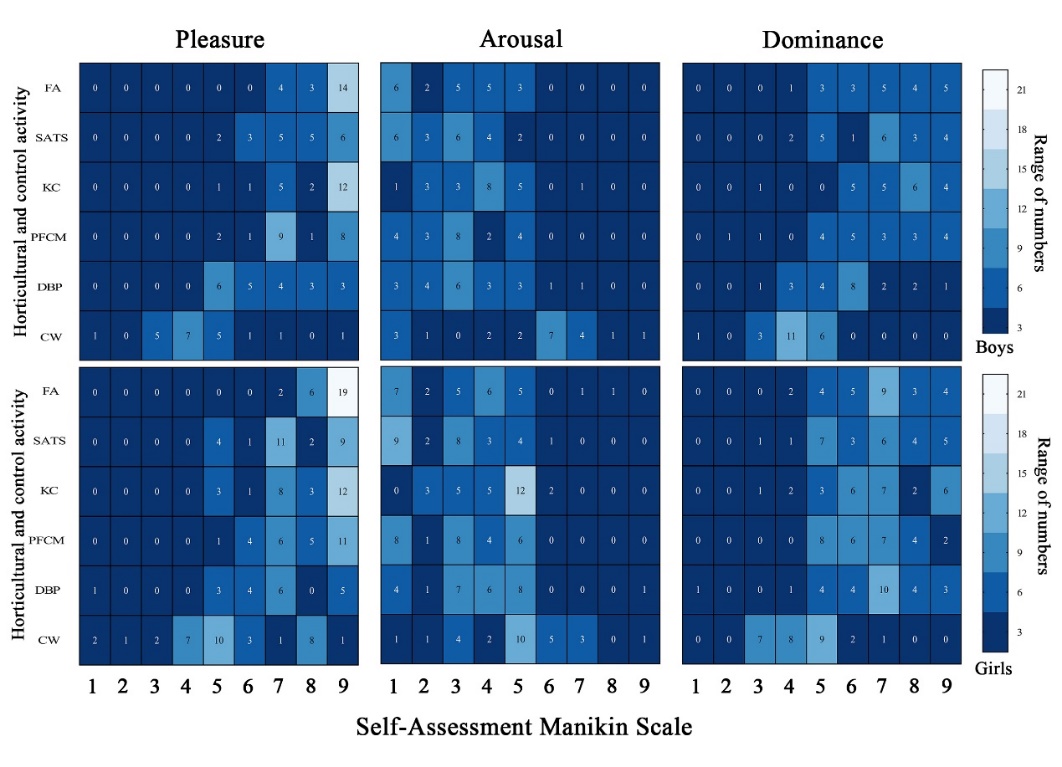
FA: Flower Arrangement, SATS: Sowing And Transplanting Seeding, KC: Kokedama Crafting, PFCM: Pressed Flower Card Making, DBP: Decorative Bottle Painting；HR=Heart Rate, RMSSD= Root Mean Square of Successive Differences, LF/HF=Low Frequency Power/High Frequency Power, SI=Stress Index, PA=Positive Affect, NA=Negative Affect, M±SD=Mean ± Standard Deviation, The effect size is reported by Cohen’s *d*.

**Supplementary Figure 1.** Comparison of Gender Differences in the Three Dimensions of the SAM.
