# Supplementary material for: Which horticultural activities are more effective for children’s recovery from stress and mental fatigue? A quasi-experimental study
Source: Front Psychol. 2024 Apr 12;15:1352186. doi: 10.3389/fpsyg.2024.1352186 (PMC11050040; doi:10.3389/fpsyg.2024.1352186)
Supplement: Supplementary file 2 [file Data_Sheet_1.PDF]

How did you feel right now?

Dear child:

We want to know how you feel about the activity you just did. Read the instructions carefully before marking your choices. Please circle the numbers that best represent your emotions and feelings. You don't have to worry about any of your chose affecting your grades or performance in school. Your responses will be kept confidential. Thank you for your participation sincerely.

Tick Requirements:

Based on how you truly felt during the activity just now. Read each adjective word and then circle the appropriate number next to the words that best represent your emotional state, i.e., to what extent did you experience the emotions described by the adjectives listed in the table?

Each adjective describing an emotion has five options: "**very slightly or not at all**" means that the emotion rarely or never occurs; "**a little**" means that the emotion or feeling occurs infrequently; "**moderately**" means that the emotion occurs occasionally; "**quite a bit**" means that the emotion occurs more frequently; and "**extremely**" means means the feeling is often prevalent in your emotions. Please circle (○) the number that best describes your first feeling from the 30 examples of adjectives that express feelings (or emotions) listed in the table. There is only one number chosen for each adjective word. Please check carefully to make sure you didn't leave out any words.

(Note: Before the activity, the research assistants explained the " Tick Requirements " and the exact meaning of the adjectives in the scale to the children in child language that ensured they fully understood.)

Feelings and Emotions

(Positive and Negative Affect Scale for children, PANAS-C)

(The actual questionnaire shows "How did I just feel right now?", not the scale headline.)

**Feeling or emotion**	**Very slightly or not at all**	**A little**	**Moderately**	**Quite a bit**	**Extremely**
Interested	1	2	3	4	5
Sad	1	2	3	4	5
Frightened	1	2	3	4	5
Alert	1	2	3	4	5
Excited	1	2	3	4	5
Ashamed	1	2	3	4	5
Upset	1	2	3	4	5
Happy	1	2	3	4	5
Strong	1	2	3	4	5
Nervous	1	2	3	4	5
Guilty	1	2	3	4	5
Energetic	1	2	3	4	5
Scared	1	2	3	4	5
Calm	1	2	3	4	5
Miserable	1	2	3	4	5
Jittery	1	2	3	4	5
Cheerful	1	2	3	4	5
Active	1	2	3	4	5
Proud	1	2	3	4	5
Afraid	1	2	3	4	5
Joyful	1	2	3	4	5
Lonely	1	2	3	4	5
Mad	1	2	3	4	5
Fearless	1	2	3	4	5
Disgusted	1	2	3	4	5
Delighted□	1	2	3	4	5
Blue	1	2	3	4	5
Daring	1	2	3	4	5
Gloomy	1	2	3	4	5
Lively	1	2	3	4	5
