# Supplementary material for: Which horticultural activities are more effective for children’s recovery from stress and mental fatigue? A quasi-experimental study
Source: Front Psychol. 2024 Apr 12;15:1352186. doi: 10.3389/fpsyg.2024.1352186 (PMC11050040; doi:10.3389/fpsyg.2024.1352186)
Supplement: Supplementary file 3 [file Data_Sheet_2.PDF]

How did I feel when doing the activity?

Dear child:

How did you feel during this activity? We want to know your state during the activity operating stage regarding your happiness, mood, and confidence (or performance). Please tick the numbers that best represent your emotions and feelings. You don't have to worry about any of your chose affecting your grades or performance in school. Your responses will be kept confidential. Thank you for your participation sincerely🌺!

Tick Requirements：

Each line of the following figures represents a state. You can imagine the cartoon characters in the figure as yourself. The first line represents the level of pleasure, so look closely at the character's facial expression **(the shape of the mouth changes)**. The character gradually changes from a frowning, depressed state on the far left to a highly delighted, smiling state on the far right.The second line represents the emotional level, so please pay particular attention to the **character's face** (eyes👀) and **internal changes**. The character gradually changes from a calm, almost sleepy state on the far left to an extremely restless emotional state. The third line represents satisfaction and confidence in yourself. Please pay particular attention to the character's **body size**, which grows from very small on the far left to a large size on the far right. This transformation represents feeling accomplished and having increased self-confidence.

Please select the number from 1 to 9 at the bottom of the image that corresponds to your current state. **(Attention: Do not circle the cartoon character image.)**

Note: 1. For the sake of children's understanding, "emotional excitement" here stands for "arousal" and "confidence and satisfaction" for "dominance." 2. Before the activity, the research assistants explained the "Tick Requirements" and the exact meaning of the cartoon character to the children in the children's language, ensuring they fully understood. 3. The actual questionnaire shows " How did I feel when doing the activity?", not the scale headline “Self-Assessment Manikin (SAM).”

How did I feel when doing the activity?

I was extremely unhappy and really disliked today’s activity.

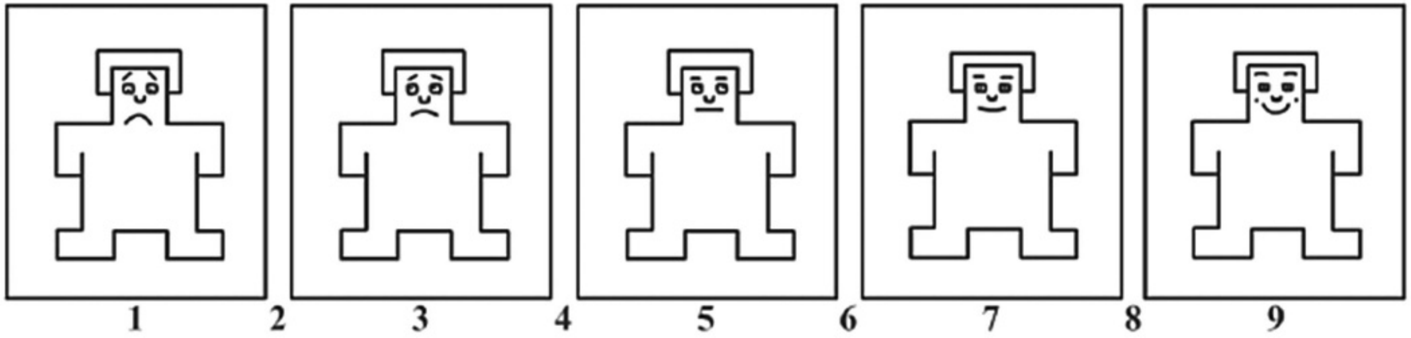

I was very happy and really enjoyed today's activity.

I was so calm and felt like falling asleep.

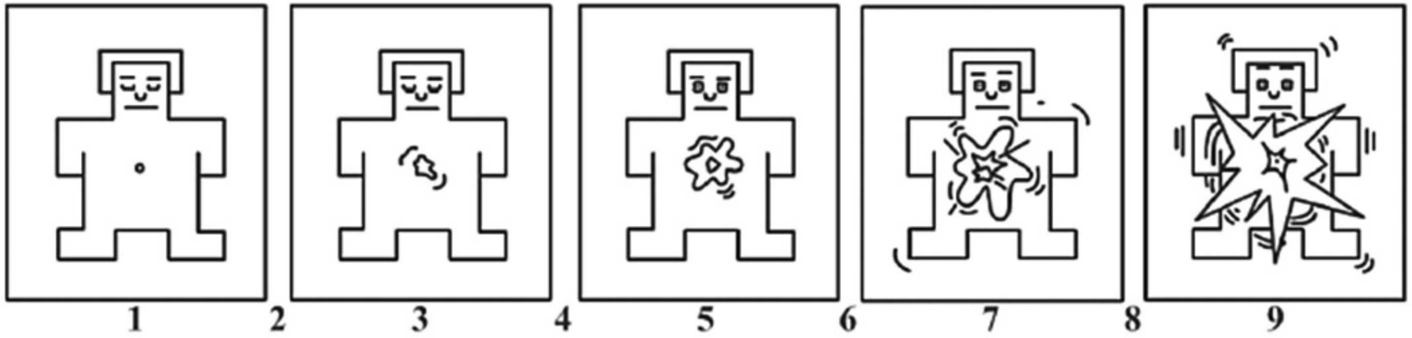

I was extremely irritable and restless; I felt like I was going to explode.

I was highly dissatisfied with the works and felt extremely unconfident.

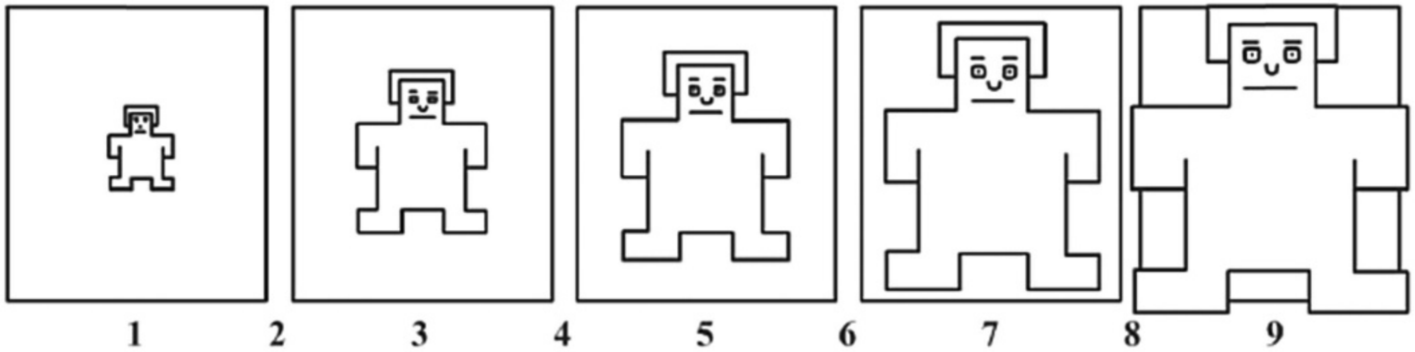

I was satisfied with my work and felt extremely confident!
