# Supplementary material for: Which horticultural activities are more effective for children’s recovery from stress and mental fatigue? A quasi-experimental study
Source: Front Psychol. 2024 Apr 12;15:1352186. doi: 10.3389/fpsyg.2024.1352186 (PMC11050040; doi:10.3389/fpsyg.2024.1352186)
Supplement: Supplementary file 4 [file Data_Sheet_3.PDF]

Study Protocol (Copy)

1 Purpose and significance

Elementary children spend most of their time in class. In the context of the "double reduction" policy (reducing school homework and off-campus or after-school training programs), strategic implementation of horticultural activity programs may help elementary school students manage stress and emotions. This study provides a scientific and practical reference for promoting elementary school horticultural activities.

2 Content of the study

2.1 Research title

Which horticultural activities are more effective for children's recovery from stress and mental fatigue? A quasi-experimental study.

2.2 Research question

(1) What physiological impact do different types of horticultural activities have on pre-stressors in elementary students?
(2) What emotional and affective impacts do different types of horticultural activities have on pre-stressors in elementary students?
(3) Are there differences in the psycho-physiological impacts of horticultural activities on elementary students?
(4) How do interactions between physiological feedback and time change during the activity while controlling for sociodemographic factors?

2.3 Subject Recruitment

The study planned to recruit subjects via snowball sampling in elementary schools in the Yangling Demonstration Zone of Agricultural High-Tech Industries, Shaanxi Province. Considering the practical abilities and comprehension of elementary students, we set the following inclusion criteria: third- to sixth-graders with normal vision and hearing, without allergies to fresh-cut flowers, and without physiological, emotional, or latent heart diseases.

2.4 Place and time

The study will be conducted during the summer mid-semester, when elementary school

students are in their normal semester condition and not stressed about final exams. The quasi-experimental design focused on a multi-purpose space in the Children's Cultural Activity Center to replicate the students' classroom. The city center location makes dropping off and picking up children easy for parents. The classroom environmental conditions will be rigorously controlled during the study.

2.5 Course contents and arrangement

The activities will be conducted as horticultural-themed courses from Monday to Saturday in the afternoons after school, with each day featuring a themed activity. Six elementary students took all courses in each session. Students participate in the daily theme activities in two-person groups. The order of group activities each day follows a rotation. After three courses, the members of each group will reorganize to ensure that each participant in the group is familiar with the others. Horticultural classes are randomized before each session, and the order will not be disclosed to participants beforehand.

The study refers to the horticultural activities classification method proposed by Park et al. (2016), excluding “outdoor gardening,” selecting the flower arrangement, *kokedama* crafting (which translates literally “moss ball” crafting), sowing and transplanting seedlings, pressed flower card-making, and decorative bottle painting with dried flowers as the representative activities for the “flower arranging art,” “live plant crafts,” “indoor planting,” “art or pressed flower crafts,” and “other activities (including food making, art, exhibitions, etc.)” categories. Each activity had unique characteristics and objectives (Table 1). We used free composition writing, a typical classroom activity, as a reference activity.

2.6 Experimental Procedure

Four researchers will conduct the experiment. One will teach the course, while the others will interpret questionnaires, wear, adjust, and remove physiological devices, maintain order, guide students after each session, and coordinate with the parents. Only the course instructor and one researcher who will monitor the devices will remain on-site after the experiment begins.

The experiment will proceed in four stages. The instructor will start the day by teaching horticultural knowledge and giving activity demonstrations (approximately 15 minutes). Students will take a brief break after the course, when two research assistants will help them put on physiological monitoring devices and make adjustments. Students will then

undergo 3 minutes of baseline monitoring. They will be given 10 minutes to complete an arithmetic test during the stress induction stages, during which a stopwatch timer will be set to increase stress. The students will complete the Positive and Negative Affect Schedule for Children (Chinese version) immediately after stress induction. The activity-operation stage will follow (20 minutes). After the activity, the students will complete the PANAS-C again, as well as the Self-Assessment Manikin (SAM) questionnaire, based on their experiences. The student's basic information (age, grade, sex, only child status, prior horticultural activity experience) and informed consent forms will be completed before the initial activity begins.

2.7 Instruments and measures

2.7.1 Physiological measures

(1) Electrocardiogram (ECG)

The proposed indicators will include heart rate, heart rate variability (HRV) (RMSSD in the time domain, and low and high-frequency ratios in the frequency domain). ECG data will be recorded in real-time using the Polar V800 with the Polar H10 chest strap (Polar Electro Oy., Kempele, Finland). The raw data will then be processed using Kubios HRV Scientific (version 3.5) software (Tarvainen et al., 2014).

(2) Electroencephalogram (EEG)

The wireless and portable NeuroSky MindWave-EEG headset (Beijing Oriental Creation Technology Co., Ltd., China) will be employed to record EEG signals from the participants' left upper frontal lobe (Fp1). The company's ThinkGearTM technology will amplify the raw EEG waves, filter out interference from electrical noise and muscle tissue movement, and transmit the preprocessed data directly to a computer via built-in microchips (Deng et al., 2020). The activity of α brain waves (8–13 Hz) will represent a person's emotional calmness and physiological relaxation, while β brain waves (14–30 Hz) will be highly correlated with alertness and effortful thinking (Qi et al., 2022). Therefore, this study will select α and β brain waves as the EEG indicators for stress feedback.

2.7.2 Psychological measures

We will use the PANAS-C and SAM, two self-reporting questionnaires, to evaluate psychological changes associated with stress and activity, which have been optimized for use in China and are widely used in children's populations(Pan et al., 2015; Shu and Ma, 2020).

The PANAS-C has positive and negative dimensions, each with 15 words that describe emotions, which are scored on a five-point scale (1 = *very slightly* or *not at all* to 5 = *extremely*) (Laurent et al., 1999).

The SAM is a nine-point picture-based assessment tool with three dimensions; the scale utilizes cartoon characters to depict diverse psychological and emotional states (Bradley and Lang, 1994). The dimensions of "pleasure," "arousal," and "dominance" will measure joy, emotional activation, and self-awareness and performance effectiveness when influenced by external stimuli, respectively.

3 Notes

Ensure participants willingly participate in this study activity and retain the freedom to withdraw at any time, for any reason. Additionally, it is vital to ensure that the guardian is informed about the experiment's specifics and has signed the informed consent document before the study commences. The guardian should be informed that the measurement data will solely be utilized for research purposes and never be disclosed for public use.

4 Reference

Bradley, M.M., and Lang, P.J. (1994). Measuring emotion: the self-assessment manikin and the semantic differential. *Journal of behavior therapy and experimental psychiatry* 25(1), 49-59.

Deng, L., Li, X., Luo, H., Fu, E.-K., Ma, J., Sun, L.-X., et al. (2020). Empirical study of landscape types, landscape elements and landscape components of the urban park promoting physiological and psychological restoration. *Urban Forestry & Urban Greening* 48, 126488.

Laurent, J., Catanzaro, S.J., Joiner Jr, T.E., Rudolph, K.D., Potter, K.I., Lambert, S., et al. (1999). A measure of positive and negative affect for children: scale development and preliminary validation. *Psychological assessment* 11(3), 326.

Pan, T., Ding, X., Sang, B., Liu, Y., Xie, S.Y., and Feng, X.Y. (2015). Reliability and Validity of the Chinese Version of the Positive and Negative Affect Scale for Children. *Chinese Journal of Clinical Psychology* 23(03), 397-400. doi: 10.16128/j.cnki.1005-3611.2015.03.004.

Park, S.-A., Lee, A.Y., Lee, G.-J., Kim, D.-S., Kim, W.S., Shoemaker, C.A., et al. (2016). Horticultural activity interventions and outcomes: A review. *Horticultural Science and Technology* 34(4), 513-527.

Qi, Y., Chen, Q., Lin, F., Liu, Q., Zhang, X., Guo, J., et al. (2022). Comparative study on birdsong and its multi-sensory combinational effects on physio-psychological restoration. *Journal of Environmental Psychology* 83, 101879.

Shu, S., and Ma, H. (2020). Restorative effects of urban park soundscapes on children's psychophysiological stress. *Applied Acoustics* 164, 107293.

Tarvainen, M.P., Niskanen, J.-P., Lipponen, J.A., Ranta-aho, P.O., and Karjalainen, P.A. (2014). Kubios HRV – Heart rate variability analysis software. *Computer Methods and Programs in Biomedicine* 113(1), 210-220. doi: https://doi.org/10.1016/j.cmpb.2013.07.024.

5 Tables

Table 1. Horticultural activities theme, contents, materials, and objectives.

Theme	Contents	Materials and tools	Objectives
Sowing and transplanting seedings (SATS)	①PPT presentation on seed morphology; Key points for seedling care and management;② Mixing soil, watering, planting seedlings, etc.	Mini sunflower seeds, edible lemon mint seedlings, cultivation soil, Nutrient-rich substrate, PE cultivation pot, watering can, plant labels	①Learn plant reproduction methods, seeding and seedling propagation techniques; ②Develop manual dexterity and hand-eye coordination; ③Experience life's growth.
Kokedama crafting (KC)	①PPT presentation on common fern growth, kokedama appreciation, and maintenance;② mixing soil, kneading soil balls, laying moss, tying ropes, and watering	*Asparagus setaceus* seedlings (7.8"-9.8" height), coarse peat moss, peat soil, spray bottle, jute twine, tweezers, tray, scissors	①Acquiring knowledge of kokedama; ②Mastering kokedama production techniques ③Experiencing the zen of kokedama;
Flower arranging (FA)	①PPT presentation on flower arrangement knowledge and work appreciation;②flower mud wrapping, branch trimming, material arrangement	Floral foam, cellophane, floral shears, floral materials, decorative bags	①Learn basic flower arranging techniques; ②Develop aesthetic, observational, and spatial perception skills.
Pressed flower card making (PFCM)	①Techniques for making pressed flower greeting cards (card layout design, tweezers handling, glue application)	Pressed flower materials, blank cards, tweezers, soft glue, markers	①Practice fine motor skills, aesthetics, and patience; ②Be grateful;
decorative bottle painting with dried flowers (DBP)	①Which plants produce dried plant materials? ②Create bottle art freely using selected dried flower materials (bow-tying at the bottle neck)	dried plant branches and fruits(cotton, lotus pods with seeds, pine cones, etc.), jute twine, acrylic paint and brushes, glass bottle	①Experience textures of dried plant materials; ②Develop imagination, aesthetics, and painting skills.

Table 2. Emotional Response Scale (1).

Dear child:

We want to know how you feel about the activity you just did. Read the instructions carefully before marking your choices. Please circle the numbers that best represent your emotions and feelings. You don't have to worry about any of your chose affecting your grades or performance in school. Your responses will be kept confidential. Thank you for your participation sincerely.

Tick Requirements:

Based on how you truly felt during the activity just now. Read each adjective word and then circle the appropriate number next to the words that best represent your emotional state, i.e., to what extent did you experience the emotions described by the adjectives listed in the table?

Each adjective describing an emotion has five options: "very slightly or not at all" means that the emotion rarely or never occurs; "a little" means that the emotion or feeling occurs infrequently; "moderately" means that the emotion occurs occasionally; "quite a bit" means that the emotion occurs more frequently; and "extremely" means means the feeling is often prevalent in your emotions. Please circle (○) the number that best describes your first feeling from the 30 examples of adjectives that express feelings (or emotions) listed in the table. There is only one number chosen for each adjective word. Please check carefully to make sure you didn't leave out any words.

Positive and Negative Affect Scale for children, PANAS-C

**Feeling or emotion**	**Very slightly or not at all**	**A little**	**Moderately**	**Quite a bit**	**Extremely**
Interested	1	2	3	4	5
Sad	1	2	3	4	5
Frightened	1	2	3	4	5
Alert	1	2	3	4	5
Excited	1	2	3	4	5
Ashamed	1	2	3	4	5
Upset	1	2	3	4	5
Happy	1	2	3	4	5
Strong	1	2	3	4	5
Nervous	1	2	3	4	5
Guilty	1	2	3	4	5
Energetic	1	2	3	4	5
Scared	1	2	3	4	5
Calm	1	2	3	4	5
Miserable	1	2	3	4	5
Jittery	1	2	3	4	5
Cheerful	1	2	3	4	5
Active	1	2	3	4	5
Proud	1	2	3	4	5
Afraid	1	2	3	4	5
Joyful	1	2	3	4	5
Lonely	1	2	3	4	5
Mad	1	2	3	4	5
Fearless	1	2	3	4	5
Disgusted	1	2	3	4	5
Delighted	1	2	3	4	5
Blue	1	2	3	4	5
Daring	1	2	3	4	5
Gloomy	1	2	3	4	5
Lively	1	2	3	4	5

Table 3. Emotional Response Scale (2).

Dear child:

How did you feel during this activity? We want to know your state during the activity operating stage regarding your happiness, mood, and confidence (or performance). Please tick (√) the numbers that best represent your emotions and feelings. You don't have to worry about any of your chose affecting your grades or performance in school. Your responses will be kept confidential. Thank you for your participation sincerely!

Tick Requirements:

Each line of the following figures represents a state. You can imagine the cartoon characters in the figure as yourself. The first line represents the level of pleasure, so look closely at the character's facial expression (the shape of the mouth changes). The character gradually changes from a frowning, depressed state on the far left to a highly delighted, smiling state on the far right.The second line represents the emotional level, so please pay particular attention to the character's face and internal changes. The character gradually changes from a calm, almost sleepy state on the far left to an extremely restless emotional state. The third line represents satisfaction and confidence in yourself. Please pay particular attention to the character's body size, which grows from very small on the far left to a large size on the far right. This transformation represents feeling accomplished and having increased self-confidence.

Please select the number from 1 to 9 at the bottom of the image that corresponds to your current state.

The Self-Assessment Manikin (SAM)

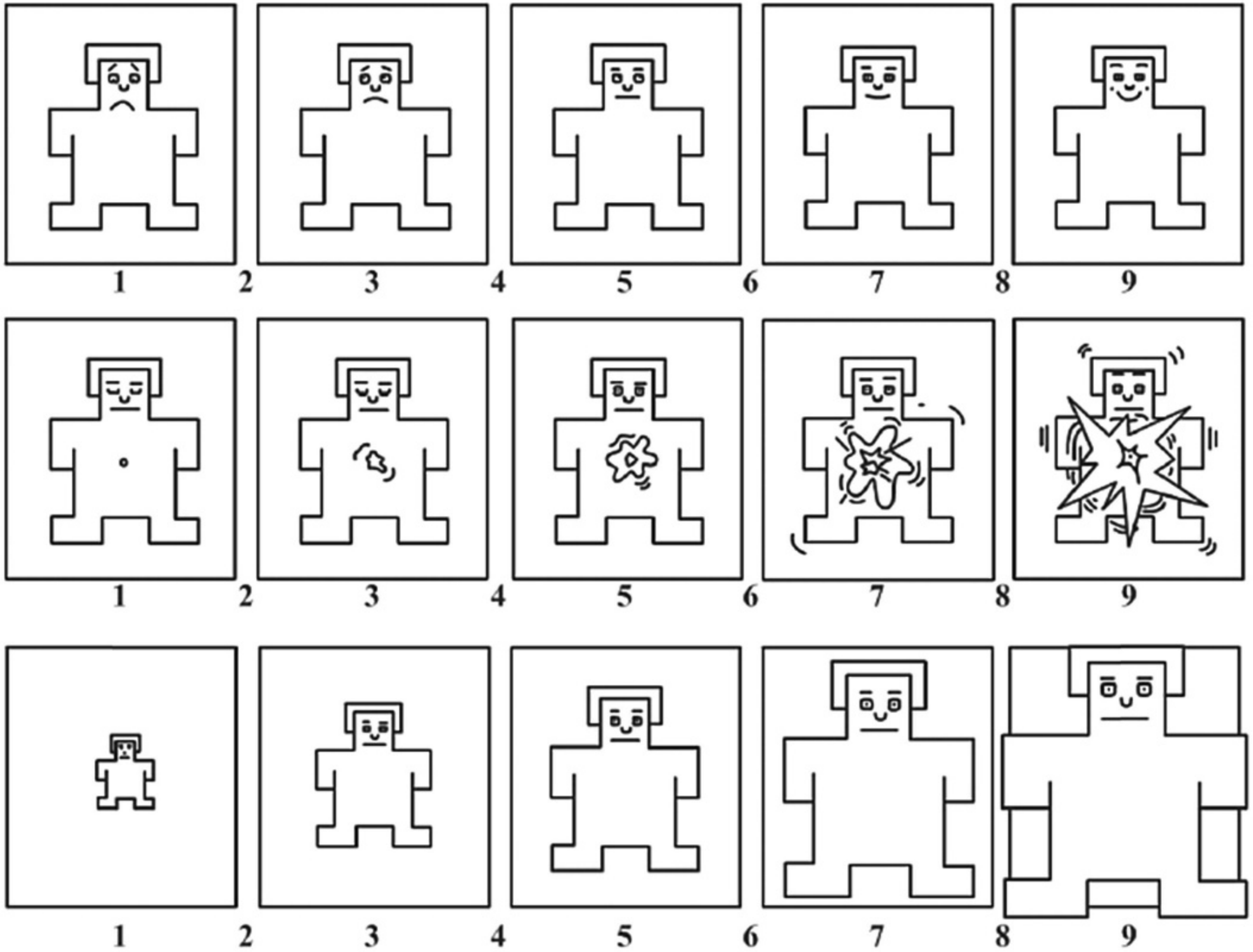

I was extremely unhappy and really disliked today's activity. — I was very happy and really enjoyed today's activity.

I was so calm and felt like falling asleep. — I was extremely irritable and restless; I felt like I was going to explode.

I was highly dissatisfied with the works and felt extremely unconfident. — I was satisfied with my work and felt extremely confident!

6 Approval Certification

Study Protocol Approval Certification

After a meticulous examination by the Ethics Committee of the College of Landscape Architecture and Art at Northwest A&F University, it has been determined that the study protocol("Which horticultural activities are more effective for children's recovery from stress and mental fatigue? A quasi-experimental study.") aligns with the principles articulated in the Declaration of Helsinki. Consequently, the experimental research work has received official approval to proceed.

hereby to certify.

the Ethics Committee of College of Landscape Architecture and Arts,
Northwest A&F University
December 10, 2021

风景园林艺术学院
